# Interleaved TMS/fMRI shows that threat decreases dlPFC-mediated top-down regulation of emotion processing

**DOI:** 10.1038/s44277-024-00007-8

**Published:** 2024-04-24

**Authors:** Milan Patel, Marta Teferi, Hannah Gura, Abigail Casalvera, Kevin G. Lynch, Frederick Nitchie, Walid Makhoul, Yvette I. Sheline, Desmond J. Oathes, Nicholas L. Balderston

**Affiliations:** 1 Center for Neuromodulation in Depression and Stress Department of Psychiatry University of Pennsylvania Philadelphia, Philadelphia, PA USA; 2 Department of Psychiatry University of Pennsylvania Philadelphia, Philadelphia, PA USA; 3 Penn Brain Science, Translation, Innovation, and Modulation Center, University of Pennsylvania, Philadelphia, PA USA; 4 Center for Brain Imaging and Stimulation, Department of Psychiatry, University of Pennsylvania, Philadelphia, PA USA

**Keywords:** Prefrontal cortex, Cognitive control

## Abstract

The dorsolateral prefrontal cortex (dlPFC) is thought to be a key site in the brain’s cognitive control network, supporting cognitive processes like attention and working memory [1–7]. There is also evidence that the dlPFC is engaged during anxiety regulation tasks, suggesting that anxiety regulation may be mediated in part by dlPFC activity [8–15]. However, the degree to which these two domains of processing overlap is unclear. Therefore, in the current study, we tested the hypothesis that the dlPFC regulates brain regions critical for the expression of anxiety. To do so, we used interleaved TMS/fMRI to record TMS-evoked BOLD responses during periods of threat compared to periods of safety. We hypothesized that TMS pulses would reduce activity in anxiety expression regions during threat. Forty-four healthy controls (no current or history of psychiatric disorders) were recruited to take part in a broader study. Participants completed the neutral, predictable, and unpredictable (NPU) threat task while receiving TMS pulses to either the right dlPFC or a control region. A whole brain analysis identified regions showing significant BOLD responses evoked by dlPFC stimulation. We then extracted these responses and compared those evoked during safe blocks to those evoked during unpredictable threat. We found that responses in the left insula (LI), right sensory/motor cortex (RSM), and a region encompassing the bilateral SMA regions (BSMA) showed significantly different responses during the safe blocks compare to the threat. During the safe periods, these regions showed significant BOLD deactivations. These deactivations were reduced during the threat blocks. Overall, these findings are largely consistent with the hypothesis that the dlPFC plays a role in the top-down control of emotion and suggest that dlPFC activity reduces downstream activity in emotional expression regions, but that this effect is reduced under threat.

## Lay summary

The dorsolateral prefrontal cortex (dlPFC) is increasingly being targeted with transcranial magnetic stimulation (TMS) to reduce anxiety; however, there is little mechanistic evidence linking dlPFC activity to specific changes in anxiety networks. Thus, the current objective was to stimulate the dlPFC noninvasively using TMS, while simultaneously recording brain activity using fMRI. We found that dlPFC stimulation reduced activity in downstream regions associated with anxiety, but that this reduction was partially blocked when people were anxious.

## Introduction

The dorsolateral prefrontal cortex (dlPFC) is thought to be a key site in the brain’s cognitive control network, supporting cognitive processes like attention and working memory [1–7]. The dlPFC is also engaged during anxiety regulation tasks, suggesting that anxiety regulation is mediated in part by dlPFC activity [8–15]. However, the degree to which these two domains of processing overlap is unclear. There is evidence that engaging in working memory processes can reduce anxiety levels during unpredictable threat [16] and there are parallel data showing that activation of the dlPFC during unpredictable threat both supports task performance [17] and is correlated with lower anxiety levels [18]. Finally, there is evidence to suggest that individuals with anxiety disorders have deficits in both working memory [19, 20] and emotion regulation [21]. Together, these data suggest that anxiety regulation may be a direct consequence of increases in dlPFC activity; however, this has yet to be experimentally evaluated. Additionally, there is evidence from clinical neuromodulation studies suggesting the opposite, namely that decreasing dlPFC activity, specifically right dlPFC activity, may reduce anxiety levels [22]. Supporting this idea, 1 Hz stimulation to the right dlPFC, which is thought to decrease cortical excitability, has been shown to reduce anxiety in individuals with comorbid depression and anxiety [23]. However, the mechanism is unclear.

One approach to experimentally testing such a hypothesis is to externally stimulate the dlPFC using noninvasive brain stimulation techniques like transcranial magnetic stimulation (TMS) [24]. TMS uses rapidly alternating magnetic fields to generate a localized electric (e)-field capable of generating action potentials in the cortex directly below the TMS coil [25–27]. By combining TMS with simultaneous measures of behavior or brain activity, it is possible to experimentally test the effect of cortical stimulation on behavior or downstream brain activity [28]. Recent advances in TMS coil and MRI pulse sequence design have made it possible to interleave TMS pulses with functional MRI sequences and record TMS-evoked BOLD responses [29]. This approach has been used previously to demonstrate patterns of downstream network activity evoked by cortical stimulation [29, 30]. Namely, stimulating regions of the cortex that show functional connections with specific sub-cortical regions, as recorded with resting state fMRI, leads to increases in BOLD activity in those regions [29–31]. Such an approach allows researchers to causally map the networks associated with the stimulation site [29, 30, 32, 33].

In the current study, we evaluated the hypothesis that the right dlPFC regulates activity in brain regions that are critical for the expression of anxiety. Participants completed the neural, predictable, unpredictable (NPU) threat task while undergoing TMS/fMRI [18, 34, 35]. During this task, participants were instructed to rate their anxiety during periods of predictable and unpredictable threat. Intermittent single TMS pulses were delivered to either the right dlPFC or a control region during counterbalanced safe and threat blocks. We hypothesized that if we stimulate the right dlPFC with TMS during the threat periods, we would observe TMS-induced decreases in BOLD activity (i.e. BOLD deactivations) in regions important for anxiety expression, like the amygdala or insula. We chose the right dlPFC because we have previous data linking right dlPFC activity to anxiety expression and because the right dlPFC is a common treatment target for patients with anxiety symptoms.

## Materials and methods

### Participants

Sixty-eight right-handed participants between the ages of 18 and 50 were recruited from Philadelphia, PA, to take part in the broader study (K01MH121777 [NLB]). A total of 44 participants elected to complete the optional TMS/fMRI visit described below. A total of 41 participants completed all aspects of the study needed for the current work (31 females, 13 males, mean age =  25.39 years, SD  =  6.55), and were included in the final analysis. A supplemental control analysis was conducted on a subset of participants (*N* = 32) who had additional TMS/fMRI data from a control site. These data are included in the Supplement. Exclusion criteria included: current or past Axis I psychiatric disorder(s) as identified with the Structured Clinical Interview (SCID) for DSM-V (Research Version) [36], use of psychoactive medications, any significant medical or neurological problems (e.g. cardiovascular illness, respiratory illness, neurological illness, seizure, etc.), and any MRI/TMS contraindications (e.g. implanted metal, history of epilepsy or seizure, etc.). For a complete list, see: www.clinicaltrial.gov (Identifier: NCT03993509). Three participants were excluded due to technical issues. All participants signed an informed consent form, and the protocol was approved by the Institutional Review Board for human subject research at the University of Pennsylvania. All procedures contributing to this work were completed in compliance with the ethical standards of the relevant national and institutional committees on human experimentation and with the Helsinki Declaration of 1975, as revised in 2008.

### Materials

#### Sternberg WM task (Targeting Visit)

Participants were presented with a series of maintain and sort trials. Each trial started with an instruction prompt to indicate the trial type, followed by a series of 5 letters, presented sequentially. Participants retained these items in working memory for a brief retention interval. On “maintain” trials, participants were instructed to remember the letters in the order presented. On “sort” trials, participants were instructed to rearrange the letters in alphabetical order. Afterward, participants were given a forced choice prompt that consisted of a letter/number combination. They were instructed to indicate whether the position of the letter in the series matched the number. Half of the trials were matches and the other half were mismatches. The duration of the letter series (1.5–2.5 s), retention interval (6.5–8.5 s), and ITI (5–8 s) were jittered across trials. The duration of the instructions (1 s) and response prompt (3 s) were fixed. There were twelve trials each for the sort and maintain conditions.

#### NPU task (TMS/fMRI Visit)

**Fig. 1:**
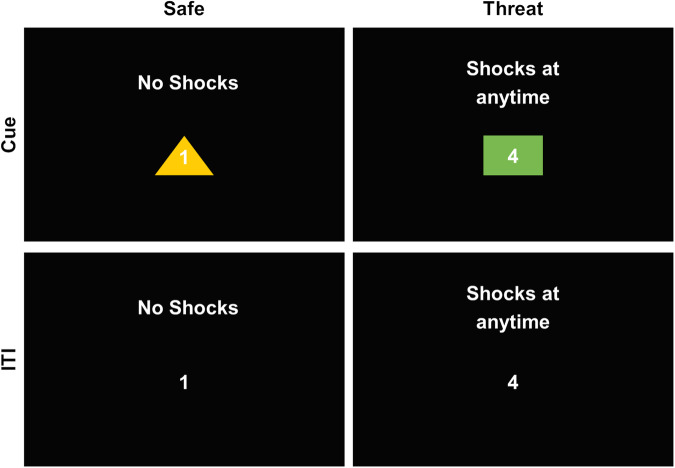
**Example screenshots from the NPU task**. We extracted data from safe and (unpredictable) threat periods during the NPU paradigm. During the safe period, participants could not receive a shock. During the threat periods, participants were instructed that they could receive a shock at any time. Instructions were visible at the top of the screen through the blocks. Periodically, a visual cue would be presented in the center of the screen. Cues would be separated by a variable intertrial interval (ITI). Participants also rated their anxiety level (from 0 to 10) throughout the task using a button box, which controlled a number in the center of the screen. The number updated in real-time. TMS pulses would be presented once during each cue (onset was jittered), and at random points during the ITI. Responses were collapsed across cue and ITI trials.

Participants completed two runs of the NPU task for each stimulation site during the TMS/fMRI session. Each run consisted of alternating blocks of neutral, predictable, and unpredictable conditions. During the neutral blocks, participants were at no risk of being shocked. During the predictable blocks, participants were only at risk of shock during the visual cue. During the unpredictable blocks, participants were at risk of shock throughout. Threat blocks were always separated by a neutral block, resulting in the following block orders: NPNUNUNP or NUNPNPNU. During these blocks, we measured the TMS-evoked BOLD response during both the presence (cue trials) and absence (intertrial interval [ITI]) of a visual cue (Fig. 1). These cues were simple colored shapes that varied across conditions. We timed the delivery of the TMS pulses to broadly replicate the timing of the white noise probes used to test the acoustic startle reflex in laboratory versions of the NPU task [18, 35]. To ensure that all conditions had the same number of trials per run (*N* = 8), there were twice as many trials per block in the threat conditions as there were in the safe condition. Each neutral block included 2 trials per condition, while each threat block included 4 trials per condition, totaling to 8 trials per condition per run. Three shocks were presented during each run at random points during either the cue (predictable condition) or the ITI (unpredictable condition) trials. Throughout the task participants rated their anxiety from a scale of 0 (feeling not anxious) to 10 (feeling extremely anxious) using an onscreen numerical scale.

#### Shock

The shock stimulus consisted of a 100 ms train of 2 ms pulses delivered at 200 Hz delivered using a DS7A constant current stimulator (Digitimer #DS7A, Ft. Lauderdale, FL). Shocks were administered to the participant’s left wrist via disposable 11 mm Ag/AgCl electrodes (Biopac Item number EL508; Goleta, CA), spaced ~2 cm apart. We calibrated the shock intensity prior to the TMS/fMRI session using an individualized workup procedure where participants rated a series of shocks shock on a scale from 1 (not uncomfortable) to 10 (uncomfortable but tolerable) until the participant reached their “level 10”. Shocks during the session were delivered at their level “10”.

#### Scans

MRI data were acquired using a 3 Tesla Siemens Prisma scanner. Structural scans were collected during the targeting session using a 64-channel head coil (Erlangen, Germany). Structural scans included a T1-weighted MPRAGE (TR = 2200 ms; TE = 4.67 ms; flip angle = 8°) with 160, 1 mm axial slices (matrix = 256 × 256; field of view (FOV) = 240 mm × 240 mm), and a T2-weighted image (TR = 3200 ms; TE = 563 ms; flip angle = variable) with 160, 1 mm sagittal slices (matrix = 256 mm × 256 mm; FOV = 240 mm × 240 mm). TMS/fMRI scans were collected using a single-channel birdcage coil (RAPID quad T/R single channel; Rimpar, Germany). Each run included 233 whole-brain BOLD images (TR = 2000 ms; TE = 30 ms; flip angle = 75°) comprised of 32, 4 mm axial slices (matrix = 64 × 64; FOV = 192 mm × 192 mm) aligned to the AC-PC line.

#### TMS/fMRI pre-processing

TMS/fMRI data were processed using the afni_proc.py script distributed with the AFNI software package [37]. The data were preprocessed using the following standard preprocessing blocks: tshift, align, tlrc, volreg, blur, mask, scale, regress corresponding to: (1) the images were slice time corrected, (2) aligned to the T1 data using a Local Pearson Correlation cost function, (3) normalized to the MNI152_2009 template distributed with AFNI, (4) individual volumes were registered to the image with the fewest outliers, (4) images were resampled to 3 mm isotropic voxels and blurred with a 6 mm Gaussian kernel, (5) masked using the union of the EPI brain mask and the skull-stripped T1, (6) scaled so that the mean of each voxel timeseries was 100. The first 4 TRs and TRs with greater than 0.5 mm displacement or greater than 30% of voxels registered as outliers were censored/scrubbed from the timeseries prior to the GLM. The participant-level GLM included a set of polynomial regressors to model the baseline and regressors of no interest corresponding to the 6 primary motion vectors and their derivatives. NPU events were modeled with variable duration blocks to account for jittering in the timing of the events. TMS pulses were modeled using an impulse response function.

#### Target localization

**Fig. 2:**
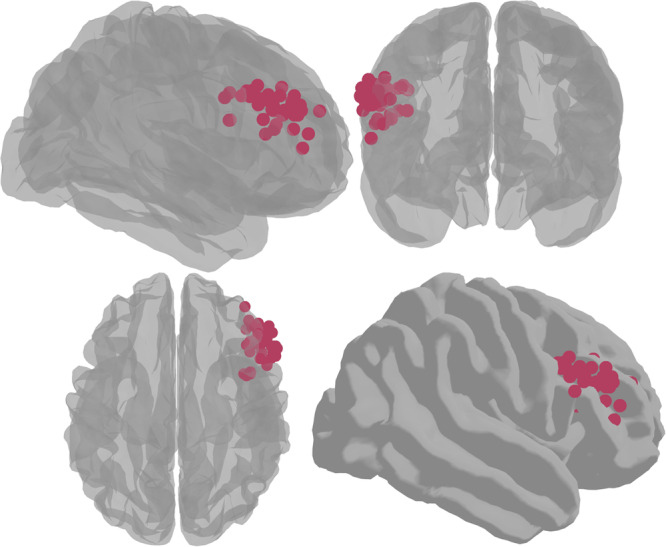
**Single participant targets in MNI space**. Spheres represent the single-participant peaks for WM-related activity during the Sternberg WM task, which were used as the TMS targets during the TMS/fMRI scans.

Data from the Sternberg WM task were used to identify the right dlPFC target coordinates for the TMS/fMRI session [34, 38]. BOLD maps from the retention interval were masked with a group-level ROI of the right dlPFC a previously published Sternberg WM dataset [25, 39]. Single participant BOLD activity was contrasted across sort and maintain trials and the coordinates for the peak voxel within this mask was extracted and used as a target (Fig. 2). We used the Brainsight (Rogue Research Inc, Montreal, Canada) frameless stereotaxic neuronavigation system to mark the target site on a swim cap worn during the TMS/fMRI session.

#### Motor threshold determination

We used a Magventure MagPro X100 stimulator with a B65 figure-8 coil to obtain resting motor threshold on the first day of stimulation. Motor threshold was determined from EMG recordings of the first dorsal interosseous muscle (FDI) using the adaptive parameter estimation by sequential testing (PEST) algorithm [40]. Stimulator output during the TMS/fMRI session was adjusted to account for differences in coil output using an in-house algorithm to determine corresponding stimulation levels between the MV B65 and MV MRI-B91 coils.

#### Stimulation

A Magventure MagPro X100 stimulator with a B91 figure-8 coil was used for the TMS/fMRI session. Periodically during the NPU task, participants received single 3-pulse 50 Hz bursts at 100% of motor threshold adjusted for differences in coil output. We timed the delivery of the TMS pulses to broadly replicate the timing of the white noise probes used to test the acoustic startle reflex in laboratory versions of the NPU task [18, 35].

### General procedure

#### General

Participants were enrolled in a broader study where they received multiple sessions of active or sham continuous Theta Burst Stimulation (cTBS) or intermittent Theta Burst Stimulation (iTBS), followed by a series of cognitive and behavioral tests. The protocol included a consent visit, a targeting visit, a TMS/fMRI visit, and several active/sham TBS visits followed by a corresponding testing session. The aims of the broader study were to examine the effect of active or sham cTBS or iTBS on anxiety and working memory. However, the procedures and results of this broader study are described elsewhere (K01MH121777 [NLB]) [38], and will not be discussed here. In the following sections, we will briefly describe procedures relevant to the data presented.

#### Consent visit

During the consent visit, participants completed a consent form, MRI safety form, TMS adult safety screen (TASS), and medical history questionnaire. Participants were also given the SCID, Montgomery-Asberg Depression Rating Scale (MADRS) [41], and an eligibility checklist, all administered by a research coordinator. Participants who met screening criteria completed a demographics questionnaire, the State/Trait Anxiety Inventory (STAI) [42], and the Beck Anxiety Inventory (BAI) [43].

#### Targeting visit

During the targeting visit, MRI scans were collected and used to identify the site and orientation of stimulation for the TMS/fMRI session. Participants were escorted to the scanner and given ear plugs, a button box, an emergency squeeze ball, and padding to minimize head movement. Next, we collected a T1, and a T2 scan. Afterward, participants completed 1 run of the Sternberg WM task and 2 resting state runs.

#### TMS/fMRI visit

During the TMS/fMRI visit, participants had their heads registered with their MRIs in Brainsight and their stimulation sites were marked on swim caps. Afterward, participants completed the shock workup procedure and were escorted to the scanner. In the scanner, the TMS coil was positioned over the right dlPFC stimulation site and the articulating arm was supported with additional padding. Participants then completed 2 runs of the NPU task with TMS delivered to the right dlPFC.

## Results

### Ratings

**Fig. 3:**
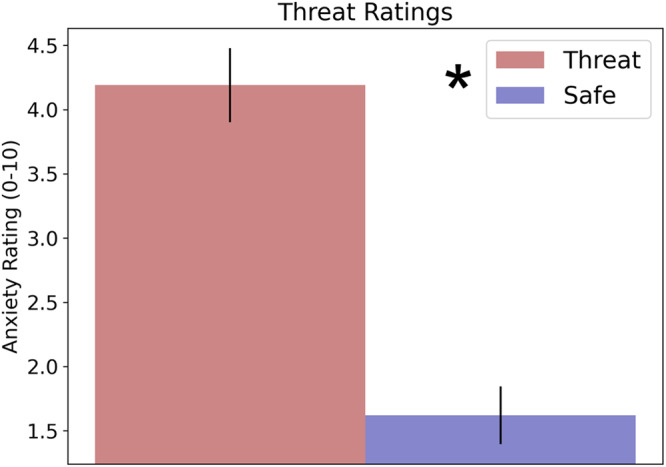
**Anxiety ratings threat task**. Anxiety ratings reported on a scale from 0 to 10. Bars represent the mean ± SEM. *p < 0.05.

Anxiety ratings at the onset of each TMS burst were extracted and averaged across all trials in the safe and threat conditions (Fig. 3). These responses were then compared using a paired sample *t*-test. As a manipulation check, we compared the anxiety ratings in safe compared to threat blocks using a *t*-test. As expected, participants reported significantly greater anxiety during threat periods compared to safe periods (t (39) = 8.85; *p* < 0.001; *d* = 1.4).

### TMS-evoked responses

We began by defining a set of regions that were directly activated by the right dlPFC TMS pulses by collapsing across conditions and computing a voxelwise *t*-test against zero. The first-level beta coefficients were extracted for all dlPFC-targeted TMS bursts. To identify TMS-evoked responses, we compared these betas to an implicit baseline using a single-sample *t*-test against 0 (3dttest++). We used cluster thresholding and *monte carlo* simulations to correct for multiple comparisons implemented by 3dClustSim [44]. We ran 10,000 Monte Carlo simulations with a voxelwise *p*-value of 0.001, a non-Gaussian (i.e. autocorrelation function) [45] estimation of the smoothness of the BOLD data, and extracted clusters comprised of voxels with adjoining faces or edges. Based on these simulations, we selected a minimum cluster size of 40, 3-mm voxels, which corresponded to a cluster-level *p*-value < 0.01.

**Fig. 4:**
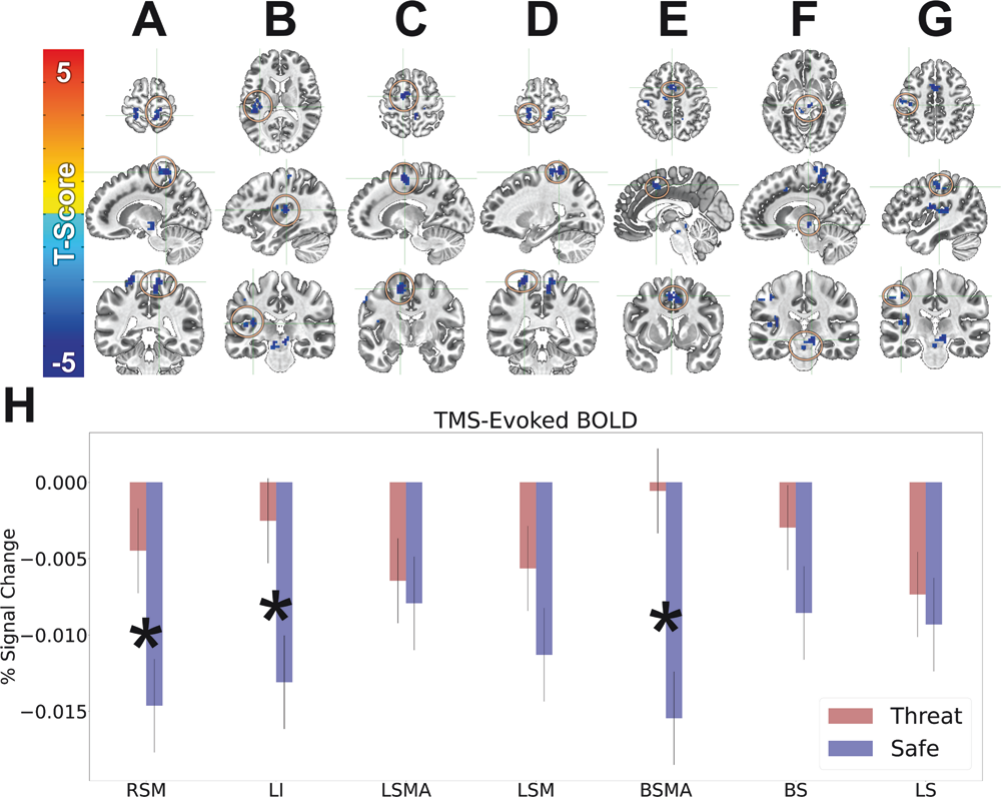
**BOLD responses evoked by TMS pulses delivered to the right dlPFC. A–G** Regions showing TMS-evoked BOLD responses that significantly differ from an implicit baseline. **H** BOLD responses in these regions plotted as a function of safe and threat conditions. RSM Right Sensory/Motor. LI Left Insula. LSMA Left SMA. LSM Left Sensory/Motor. BSMA Bilateral SMA. BS Brainstem. LS Left Sensory. Warm colors represent activations. Cool colors represent deactivations. Bars represent the mean ± SEM. *p < 0.05.

We identified 7 clusters/regions with TMS-evoked responses that significantly differed from zero (Fig. 4A–G, Table 1). To determine whether the TMS-evoked responses in these regions differed as a function of threat, we extracted the average dlPFC-targeted TMS-evoked BOLD response during the safe and threat conditions for each of the clusters identified in the whole brain analysis. We then compared these responses using a paired sample *t*-test. We repeated this process for our control site evoked responses in a subset of individuals who also had data targeting the right IPS. We found that TMS-evoked responses in the right sensory/motor cortex (Fig. 4H “RSM”), the left insula (Fig. 4H “LI”), and a region encompassing the bilateral SMA regions (Fig. 4H “BSMA”) differed significantly in the threat compared to safe conditions. Across all 3 regions, there was a significant TMS-evoked BOLD deactivation in the safe periods that was significantly attenuated in the threat periods (RSM: *t* (40) = 2.29; *p* = 0.027; *d* = 0.36; LI: *t* (40) = 2.27; *p* = 0.029; *d* = 0.37; BSMA: *t* (40) = 2.25; *p* = 0.03; *d* = 0.36). No other region showed a significant differentiation as a function of threat (all *p*-values > 0.05).

**Table 1:** Demographic information.

Measure	Value
*Recruitment*	
Started	11/8/2019
Ended	10/26/2022
Age	25.18 (6.04)
*Race*	
Asian	17.86%
White	58.93%
Black or African American	19.64%
More than one Race	1.79%
Other	1.79%
*Ethnicity*	
Hispanic	7.14%
*Sex*	
Male	30.36%
Female	69.64%
*Questionnaire Data*	
TAI	27.52 (6.18)
SAI	26.88 (4.87)
BAI	2.43 (3.64)
*Task Details*	
MT (% Output)	59.79 (9.98)
e-field (V/M)	62.22 (13.4)
Shock (mA)	3.23 (3.01)

## Discussion

In this study, we investigated the effect of an experimental manipulation of anxiety/threat on TMS-evoked BOLD responses targeted at the right dlPFC. By administering TMS pulses interleaved with the fMRI acquisition, we were able to measure BOLD responses downstream from the dlPFC stimulation site. By nesting these TMS pulses in alternating periods of safety and threat, we were able to determine how threat effected activity in regions downstream from the dlPFC. During safe periods, we found that right dlPFC stimulation led to BOLD deactivation in a variety of regions across several brain networks. However, during the threat periods, this BOLD deactivation was reduced (i.e. responses were less negative) in regions like the anterior insula that have been highlighted in many prior studies of anxiety and threat processing [18, 46]. Together these results suggest that the dlPFC plays a broad role in top-down suppression across multiple networks, which may filter distracting information out of working memory. However, during periods of elevated threat and arousal, this top-down suppression is reduced, perhaps allowing for increased vigilance to threats in the environment.

The dlPFC is known to play a key role in both working memory processes and emotion regulation [47]. However, the link between these roles is currently unclear. Recently, we proposed a model suggesting that the primary role of the dlPFC is to subserve working memory related functions like the maintenance, manipulation, and suppression of items in short term stores [18, 20, 34, 38]. Furthermore, we proposed that the left and right dlPFC were specialized to process distinct domains of content in this processing [19, 47]. According to this model, the left dlPFC is specialized for verbal content, while the right dlPFC is specialized for non-verbal content. Accordingly, the left and right dlPFC could potentially play distinct roles in emotion regulation, with the left mediating effortful verbal regulation strategies, and the right mediating more automatic non-verbal strategies. The current results fit within this model as external stimulation of the right dlPFC decreased BOLD activity in emotion-related regions. This is consistent with the hypothesis that the right dlPFC activity may automatically regulate emotion by suppressing activity in emotion-related regions during safe periods and to a lesser extent during threat periods. However, given that we did not stimulate the left dlPFC, we have no data to suggest that these results are isolated to the right dlPFC. Future studies should directly test the effects of right vs. left dlPFC stimulation on activity in downstream regions important for emotional expression.

Our results offer preliminary evidence supporting the hypothesis that the dlPFC is involved in the top-down regulation of anxiety. We further raise the testable hypothesis that impairment of this top-down control of anxiety may potentially exacerbate some of these core components (e.g. hyperarousal, hypervigilance, impaired attention control, and overgeneralization, etc.) in individuals with anxiety disorders. Clinical anxiety disorders encompass a constellation of symptoms including hyperarousal, hypervigilance, impaired attention control, and threat cue overgeneralization [48, 49]. Many of these symptoms could be explained using this working memory framework. For instance, hypervigilance and impaired attention control could arise from impaired top-down inhibition of distraction-related activity when cognitive demands are high [48, 50]. Similarly, overgeneralization of threat could occur when novel memory encoding events occur during periods of elevated arousal that are mediated by this impaired top-down inhibition [51, 52]. These memories, in non-anxious individuals would typically be recorded as neutral events, but in highly anxious individuals, they may be encoded with a negative valence. However, this hypothesis should be tested in future studies in patient populations.

The dlPFC is one of the most common sites for rTMS in the treatment of depression, and there is evidence to suggest that excitatory left dlPFC stimulation improves depression symptoms by normalizing connectivity between the dlPFC and the subgenual anterior cingulate cortex [20, 53, 54]. However, the mechanisms that mediate anxiety reduction following right dlPFC stimulation are less clear. Indeed, it is even unclear what type of stimulation would offer the best results. There is evidence that 1 Hz stimulation to the right dlPFC can reduce anxiety symptoms in depressed individuals. There is also some evidence that 5 Hz and iTBS to the right dlPFC can improve symptoms of PTSD [55–61]. While there is no clear-cut relationship between stimulation type and excitability, it is thought that 1 Hz is potentially inhibitory, while 5 Hz and iTBS are potentially excitatory. With opposite theorized effects it is difficult to suggest that these clinical stimulation protocols are targeting similar mechanisms [62]. In our larger project, we measured the effect of either cTBS or iTBS on anxiety potentiated startle [38]. Contrary to our hypotheses, both cTBS and iTBS increased anxiety potentiated startle, further complicating our attempts at deriving a comprehensive mechanistic explanation of the link between dlPFC stimulation and anxiety. It should be noted that our studies were conducted in low anxious healthy individuals and may not generalize to patient populations. One might expect distinct effects for the differing theta burst protocols; however, future research is needed. Suffice it to say that our current finding that external stimulation of the right dlPFC directly inhibits activity in downstream regions will be important for disentangling these effects.

Concurrent TMS/fMRI offers a unique translational perspective that is useful for understanding the networks mediating psychopathology. By experimentally stimulating a region of the brain and then directly measuring the activity evoked by this stimulation, it is possible to causally determine the downstream targets of this region. For instance, single pulses to motor cortex activate a network of regions important for motor activity, like premotor cortex, and supplementary motor area [63]. Similarly, stimulating regions of the dorsal attention network causes downstream modulation of visual circuits [63]. Single pulses to the left dlPFC evoke responses in reward regions compared to sham and control site data [64, 65]. These downstream TMS-evoked responses scale with machine output, demonstrating their validity as a measure of target engagement [65]. In addition, TMS-evoked responses are state-dependent (i.e. modulated by ongoing activity). For instance, one study found that TMS enhanced FPN activations and BOLD deactivations in the DMN during high load blocks [66].

Critically, this evoked network response can then be used to assess neuromodulatory interventions by using single pulse probe before and after a neuromodulatory course of rTMS. For example, TMS-evoked responses are also able to predict circuit-level plasticity and behavior at later timepoints, thus providing an acute measure reflecting the chronic neurobiological changes associated with clinical TMS treatment [67]. Also, within-session theta burst stimulation to cortical sites can modulate activity in downstream subcortical sites and affect subsequent behavior [68]. Similar effects are seen with rTMS. For example, right compared to left HF-rTMS is associated with greater dACC and dmPFC responses [69], and lf-rTMS can increase brain activity at the striatum, thalamus, and areas of the default mode network when applied to the right, but not to the left DLPFC [70]. Finally, TMS-evoked responses collected during unpredictable threat, show that the right dlPFC seems to regulate activity in downstream regions important to emotion processing. This BOLD deactivation is attenuated by exogenous shock threat [71].

### Strengths and limitations

By combining TMS and fMRI techniques, this study was able to concurrently (interleaved) stimulate the right dlPFC and record brain activity in functionally connected regions, providing direct evidence that right dlPFC activity can reduce activity in downstream brain areas. Despite this innovation, the study has several limitations. First, by using an active control region with potentially overlapping downstream connections, it is difficult to disentangle the effects of the target and the control site. We accounted for this by analyzing the control site data separately and showing that the pattern of results differed from those evoked by active stimulation of our dlPFC target. However, future work should incorporate the use of a realistic sham condition such as electrical stimulation of the scalp. Additionally, of the 68 initially recruited participants, only 41 completed the TMS/fMRI sessions, and because the control condition was added later, we do not have data from all participants collected from this region (*N* = 32). To prioritize the results from the dlPFC condition, the dlPFC/IPS order was not counterbalanced among participants. Therefore, the findings from the IPS control analyses are potentially subject to order effects. Future work should extend these results and test their implications in a larger sample. Finally, although we induced anxiety using threat of unpredictable shock, this paradigm is not a stand-in for clinical anxiety symptomology and pathology. Future work should include anxious participants in the sample.

## Conclusions

The current work offers a novel experimental test of the hypothesis that the dlPFC regulates emotion by reducing activity in regions associated with emotional expression and offers preliminary support for this hypothesis. Accordingly, these results add to our understanding of the mechanisms underlying the effectiveness of dlPFC stimulation protocols for anxiety reduction. Future research should expand on these findings by investigating links between TMS-evoked responses and behavior/symptom changes following neuromodulatory courses of TMS in both patients with anxiety as well as healthy controls.

## Supplementary information

Supplemental Material

## References

[1] Cieslik EC, Zilles K, Caspers S, Roski C, Kellermann TS, Jakobs O, et al. Is There “One” DLPFC in cognitive action control? Evidence for heterogeneity from co-activation-based parcellation. Cereb Cortex. 2013;23:2677–89. 22918987 10.1093/cercor/bhs256 PMC3792742

[2] Harding IH, Yücel M, Harrison BJ, Pantelis C, Breakspear M. Effective connectivity within the frontoparietal control network differentiates cognitive control and working memory. NeuroImage. 2015;106:144–53. 25463464 10.1016/j.neuroimage.2014.11.039

[3] Barbey AK, Koenigs M, Grafman J. Dorsolateral prefrontal contributions to human working memory. Cortex. 2013;49:1195–205. 22789779 10.1016/j.cortex.2012.05.022 PMC3495093

[4] Altamura M, Elvevåg B, Blasi G, Bertolino A, Callicott JH, Weinberger DR, et al. Dissociating the effects of Sternberg working memory demands in prefrontal cortex. Psychiatry Res Neuroimaging. 2007;154:103–14. 10.1016/j.pscychresns.2006.08.002 17292590

[5] Curtis CE, D’Esposito M. Persistent activity in the prefrontal cortex during working memory. Trends Cogn Sci 2003;7:415–23. 12963473 10.1016/s1364-6613(03)00197-9

[6] Geier CF, Garver KE, Luna B. Circuitry underlying temporally extended spatial working memory. NeuroImage. 2007;35:904–15. 17292627 10.1016/j.neuroimage.2006.12.022 PMC4397654

[7] Feredoes E, Heinen K, Weiskopf N, Ruff C, Driver J. Causal evidence for frontal involvement in memory target maintenance by posterior brain areas during distracter interference of visual working memory. Proc Natl Acad Sci 2011;108:17510–5. 21987824 10.1073/pnas.1106439108 PMC3198359

[8] Basten U, Stelzel C, Fiebach CJ. Trait anxiety and the neural efficiency of manipulation in working memory. Cogn Affect Behav Neurosci 2012;12:571–88. 22644759 10.3758/s13415-012-0100-3 PMC3400031

[9] Fales CL, Barch DM, Burgess GC, Schaefer A, Mennin DS, Gray JR, et al. Anxiety and cognitive efficiency: differential modulation of transient and sustained neural activity during a working memory task. Cogn Affect Behav Neurosci 2008;8:239–53. 18814461 10.3758/cabn.8.3.239

[10] Peers PV, Simons JS, Lawrence AD. Prefrontal control of attention to threat. Front Hum Neurosci 2013;7:24. 23386824 10.3389/fnhum.2013.00024 PMC3564011

[11] Nitschke JB, Sarinopoulos I, Mackiewicz KL, Schaefer HS, Davidson RJ. Functional neuroanatomy of aversion and its anticipation. NeuroImage. 2006;29:106–16. 16181793 10.1016/j.neuroimage.2005.06.068

[12] Shang J, Fu Y, Ren Z, Zhang T, Du M, Gong Q, et al. The common traits of the ACC and PFC in anxiety disorders in the DSM-5: meta-analysis of voxel-based morphometry studies. PLoS ONE. 2014;9:e93432. 24676455 10.1371/journal.pone.0093432 PMC3968149

[13] Forster S, Nunez Elizalde AO, Castle E, Bishop SJ. Unraveling the anxious mind: anxiety, worry, and frontal engagement in sustained attention versus off-task processing. Cereb Cortex. 2015;25:609–18. 24062316 10.1093/cercor/bht248 PMC4318530

[14] Bishop SJ. Trait anxiety and impoverished prefrontal control of attention. Nat Neurosci 2009;12:92–98. 19079249 10.1038/nn.2242

[15] Carter RM, O’Doherty JP, Seymour B, Koch C, Dolan RJ. Contingency awareness in human aversive conditioning involves the middle frontal gyrus. NeuroImage. 2006;29:1007–12. 16246595 10.1016/j.neuroimage.2005.09.011

[16] Balderston NL, Quispe-Escudero D, Hale E, Davis A, O’Connell K, Ernst M, et al. Working memory maintenance is sufficient to reduce state anxiety. Psychophysiology. 2016;53:1660–8. 10.1111/psyp.12726 27434207 10.1111/psyp.12726 PMC5061597

[17] Balderston NL, Hsiung A, Ernst M, Grillon C. Effect of threat on right dlPFC activity during behavioral pattern separation. J Neurosci. 2017;37:9160–71. 28842415 10.1523/JNEUROSCI.0717-17.2017 PMC5607464

[18] Balderston NL, Liu J, Roberson-Nay R, Ernst M, Grillon C. The relationship between dlPFC activity during unpredictable threat and CO2-induced panic symptoms. Transl Psychiatry. 2017;7:1266. 29213110 10.1038/s41398-017-0006-5 PMC5802456

[19] Balderston NL, Flook E, Hsiung A, Liu J, Thongarong A, Stahl S, et al. Patients with anxiety disorders rely on bilateral dlPFC activation during verbal working memory. Soc Cogn Affect Neurosci 2020;15:1288–98. 33150947 10.1093/scan/nsaa146 PMC7759210

[20] Balderston NL, Vytal KE, O'Connell K, Torrisi S, Letkiewicz A, Ernst M, et al. Anxiety patients show reduced working memory related dlPFC activation during safety and threat: research article: anxiety patients show reduced dlPFC activity. Depress Anxiety. 2017;34:25–36. 27110997 10.1002/da.22518 PMC5079837

[21] Diefenbach GJ, Assaf M, Goethe JW, Gueorguieva R, Tolin DF. Improvements in emotion regulation following repetitive transcranial magnetic stimulation for generalized anxiety disorder. J Anxiety Disord 2016;43:1–7. 27467027 10.1016/j.janxdis.2016.07.002

[22] Mantovani A, Aly M, Dagan Y, Allart A, Lisanby SH. Randomized sham controlled trial of repetitive transcranial magnetic stimulation to the dorsolateral prefrontal cortex for the treatment of panic disorder with comorbid major depression. J Affect Disord 2013;144:153–9. 22858212 10.1016/j.jad.2012.05.038

[23] White D, Tavakoli S. Repetitive transcranial magnetic stimulation for treatment of major depressive disorder with comorbid generalized anxiety disorder. Ann Clin Psychiatry. 2015;27:192–6. 26247218

[24] Lefaucheur JP, André-Obadia N, Antal A, Ayache SS, Baeken C, Benninger DH, et al. Evidence-based guidelines on the therapeutic use of repetitive transcranial magnetic stimulation (rTMS). Clin Neurophysiol. 2014;125:2150–206. 25034472 10.1016/j.clinph.2014.05.021

[25] Balderston NL, Roberts C, Beydler EM, Deng ZD, Radman T, Luber B, et al. A generalized workflow for conducting electric field–optimized, fMRI-guided, transcranial magnetic stimulation. Nat Protoc 2020;15:3595–614. 33005039 10.1038/s41596-020-0387-4 PMC8123368

[26] Thielscher A, Opitz A, Windhoff M. Impact of the gyral geometry on the electric field induced by transcranial magnetic stimulation. NeuroImage. 2011;54:234–43. 20682353 10.1016/j.neuroimage.2010.07.061

[27] Opitz A, Paulus W, Will S, Antunes A, Thielscher A. Determinants of the electric field during transcranial direct current stimulation. NeuroImage. 2015;109:140–50. 25613437 10.1016/j.neuroimage.2015.01.033

[28] Deng Z-D, Luber B, Balderston NL, Velez Afanador M, Noh MM, Thomas J, et al. Device-based modulation of neurocircuits as a therapeutic for psychiatric disorders. Annu Rev Pharmacol Toxicol 2020;60:591–614. 31914895 10.1146/annurev-pharmtox-010919-023253 PMC8100981

[29] Oathes DJ, Balderston NL, Kording KP, DeLuisi JA, Perez GM, Medaglia JD, et al. Combining transcranial magnetic stimulation with functional magnetic resonance imaging for probing and modulating neural circuits relevant to affective disorders. WIREs Cogn Sci 2021;12:e1553. 10.1002/wcs.1553 PMC8521438 33470055

[30] Oathes DJ, Zimmerman JP, Duprat R, Japp SS, Scully M, Rosenberg BM, et al. Resting fMRI-guided TMS results in subcortical and brain network modulation indexed by interleaved TMS/fMRI. Exp Brain Res 2021;239:1165–78. 33560448 10.1007/s00221-021-06036-5 PMC8521442

[31] Oathes DJ, Duprat RJP, Reber J, Liang X, Scully M, Long H, et al. Non-invasively targeting, probing and modulating a deep brain circuit for depression alleviation. Nat Ment Health. 2023;1:10–1042. 10.1038/s44220-023-00165-2 PMC12919846 41726164

[32] Chen AC, Oathes DJ, Chang C, Bradley T, Zhou ZW, Williams LM, et al. Causal interactions between fronto-parietal central executive and default-mode networks in humans. Proc Natl Acad Sci USA. 2013;110:19944–9. 24248372 10.1073/pnas.1311772110 PMC3856839

[33] Gratton C, Lee TG, Nomura EM, D’Esposito M. The effect of theta-burst TMS on cognitive control networks measured with resting state fMRI. Front Syst Neurosci 2013;7:124. 24416003 10.3389/fnsys.2013.00124 PMC3874542

[34] Balderston NL, Beydler EM, Roberts C, Deng ZD, Radman T, Lago T, et al. Mechanistic link between right prefrontal cortical activity and anxious arousal revealed using transcranial magnetic stimulation in healthy subjects. Neuropsychopharmacology. 2020;45:694–702. 31791039 10.1038/s41386-019-0583-5 PMC7021903

[35] Schmitz A, Grillon C. Assessing fear and anxiety in humans using the threat of predictable and unpredictable aversive events (the NPU-threat test). Nat Protoc 2012;7:527–32. 22362158 10.1038/nprot.2012.001 PMC3446242

[36] First MB, Williams JBW, Karg RS, Spitzer RL. Structured Clinical Interview for DSM-5—Research Version (SCID-5 for DSM-5, Research Version; SCID-5-RV). Arlington, VA: American Psychiatric Association; 2015.

[37] Cox RW. AFNI: software for analysis and visualization of functional magnetic resonance neuroimages. Comput Biomed Res 1996;29:162–73. 8812068 10.1006/cbmr.1996.0014

[38] Teferi M, Makhoul W, Deng ZD, Oathes DJ, Sheline Y, Balderston NL. Continuous theta-burst stimulation to the right dorsolateral prefrontal cortex may increase potentiated startle in healthy individuals. Biol Psychiatry Glob Open Sci 2023;3:470–9. 37519467 10.1016/j.bpsgos.2022.04.001 PMC10382694

[39] Balderston NL, Flook E, Hsiung A, Liu J, Thongarong A, Stahl S, et al. Anxiety patients rely on bilateral DLPFC activation during verbal working memory. Soc Cogn Affect Neurosci 2020;15:1–11. 10.1093/scan/nsaa146 33150947 10.1093/scan/nsaa146 PMC7759210

[40] Borckardt JJ, Nahas Z, Koola J, George MS. Estimating resting motor thresholds in transcranial magnetic stimulation research and practice. J ECT. 2006;22:169–75. 16957531 10.1097/01.yct.0000235923.52741.72

[41] Montgomery SA, Asberg M. A new depression scale designed to be sensitive to change. Br J Psychiatry. 1979;134:382–9. 444788 10.1192/bjp.134.4.382

[42] Spielgberger CD, Gorsuch RL, Lushene R, Vagg PR, Jacobs GA. Manual for the State-Trait Anxiety Inventory. Palo Alto, CA: Consulting Psychologists Press; 1983.

[43] Beck AT, Epstein N, Brown G, Steer RA. An inventory for measuring clinical anxiety: psychometric properties. J Consult Clin Psychol. 1988;56:893–7. 3204199 10.1037//0022-006x.56.6.893

[44] Forman SD, Cohen JD, Fitzgerald M, Eddy WF, Mintun MA, Noll DC. Improved assessment of significant activation in functional magnetic resonance imaging (fMRI): use of a cluster-size threshold. Magn Reson Med 1995;33:636–47. 7596267 10.1002/mrm.1910330508

[45] Cox RW, Chen G, Glen DR, Reynolds RC, Taylor PA. FMRI clustering in AFNI: false-positive rates redux. Brain Connect. 2017;7:152–71. 28398812 10.1089/brain.2016.0475 PMC5399747

[46] Fullana MA, Harrison BJ, Soriano-Mas C, Vervliet B, Cardoner N, Àvila-Parcet A, Radua J. Neural signatures of human fear conditioning: an updated and extended meta-analysis of fMRI studies. Mol Psychiatry. 2015;21:500–8. 26122585 10.1038/mp.2015.88

[47] White LK, Makhoul W, Teferi M, Sheline YI, Balderston NL. The role of dlPFC laterality in the expression and regulation of anxiety. Neuropharmacology. 2023;224:109355. 36442650 10.1016/j.neuropharm.2022.109355 PMC9790039

[48] Robinson OJ, Vytal K, Cornwell BR, Grillon C. The impact of anxiety upon cognition: perspectives from human threat of shock studies. Front Hum Neurosci 2013;7:203. 23730279 10.3389/fnhum.2013.00203 PMC3656338

[49] Kimble M, Boxwala M, Bean W, Maletsky K, Halper J, Spollen K, et al. The impact of hypervigilance: evidence for a forward feedback loop. J Anxiety Disord 2014;28:241–5. 24507631 10.1016/j.janxdis.2013.12.006 PMC4211931

[50] Eysenck MW, Derakshan N, Santos R, Calvo MG. Anxiety and cognitive performance: attentional control theory. Emotion. 2007;7:336–53. 17516812 10.1037/1528-3542.7.2.336

[51] Lissek S, Kaczkurkin AN, Rabin S, Geraci M, Pine DS, Grillon C. Generalized anxiety disorder is associated with overgeneralization of classically conditioned fear. Biol Psychiatry. 2013;75:1–7. 10.1016/j.biopsych.2013.07.025 PMC3938992 24001473

[52] Lissek S, Rabin SJ, McDowell DJ, Dvir S, Bradford DE, Geraci M, et al. Impaired discriminative fear-conditioning resulting from elevated fear responding to learned safety cues among individuals with panic disorder. Behav Res Ther 2009;47:111–8. 19027893 10.1016/j.brat.2008.10.017 PMC2758527

[53] Zhang M, Wang R, Luo X, Zhang S, Zhong X, Ning Y, et al. Repetitive transcranial magnetic stimulation target location methods for depression. Front Neurosci 2021;15:695423. 34566561 10.3389/fnins.2021.695423 PMC8458642

[54] Ge R, Humaira A, Gregory E, Alamian G, MacMillan EL, Barlow L, et al. Predictive value of acute neuroplastic response to rTMS in treatment outcome in depression: a concurrent TMS-fMRI trial. Am J Psychiatry. 2022;179:500–8. 35582784 10.1176/appi.ajp.21050541

[55] Philip NS, Barredo J, Aiken E, Larson V, Jones RN, Shea MT, et al. Theta-burst transcranial magnetic stimulation for posttraumatic stress disorder. Am J Psychiatry. 2019;176:939–48. 31230462 10.1176/appi.ajp.2019.18101160 PMC6824981

[56] Nursey J, Sbisa A, Knight H, Ralph N, Cowlishaw S, Forbes D, et al. Exploring theta burst stimulation for post-traumatic stress disorder in australian veterans—a pilot study. Mil Med 2020;185:e1770–8. 32601710 10.1093/milmed/usaa149

[57] Philip NS, Ramanathan D, Gamboa B, Brennan MC, Kozel FA, Lazzeroni L, et al. Repetitive transcranial magnetic stimulation for depression and posttraumatic stress disorder in veterans with mild traumatic brain injury. Neuromodulation Technol Neural Interface. 2023;26:878–84. 10.1016/j.neurom.2022.11.015 PMC10765323 36737300

[58] Carpenter LL, Conelea C, Tyrka AR, Welch ES, Greenberg BD, Price LH, et al. 5 Hz repetitive transcranial magnetic stimulation for posttraumatic stress disorder comorbid with major depressive disorder. J. Affect. Disord. 2018;235:414–20. 29677606 10.1016/j.jad.2018.04.009 PMC6567988

[59] Boggio PS, Rocha M, Oliveira MO, Fecteau S, Cohen RB, Campanhã C, et al. Noninvasive brain stimulation with high-frequency and low-intensity repetitive transcranial magnetic stimulation treatment for posttraumatic stress disorder. J Clin Psychiatry. 2010;71:992–9. 20051219 10.4088/JCP.08m04638blu PMC3260527

[60] Isserles M, Tendler A, Roth Y, Bystritsky A, Blumberger DM, Ward H, et al. Deep transcranial magnetic stimulation combined with brief exposure for posttraumatic stress disorder: a prospective multisite randomized trial. Biol Psychiatry. 2021;90:721–8. 34274108 10.1016/j.biopsych.2021.04.019

[61] van 't Wout-Frank M, Shea MT, Sorensen DO, Faucher CR, Greenberg BD, Philip NS. A secondary analysis on effects of theta burst transcranial magnetic stimulation to reduce anger in veterans with posttraumatic stress disorder. Neuromodulation. 2021;24:870–8. 32945055 10.1111/ner.13256 PMC8453662

[62] Di Lazzaro V, Dileone M, Pilato F, Capone F, Musumeci G, Ranieri F, et al. Modulation of motor cortex neuronal networks by rTMS: comparison of local and remote effects of six different protocols of stimulation. J Neurophysiol 2011;105:2150–6. 21346213 10.1152/jn.00781.2010

[63] Mizutani-Tiebel Y, Tik M, Chang KY, Padberg F, Soldini A, Wilkinson Z, et al. Concurrent TMS-fMRI: technical challenges, developments, and overview of previous studies. Front Psychiatry. 2022;13:825205. 35530029 10.3389/fpsyt.2022.825205 PMC9069063

[64] Hanlon CA, Canterberry M, Taylor JJ, DeVries W, Li X, Brown TR, et al. Probing the frontostriatal loops involved in executive and limbic processing via interleaved TMS and functional MRI at two prefrontal locations: a pilot study. PLoS ONE. 2013;8:1–10. 10.1371/journal.pone.0067917 PMC3706588 23874466

[65] Jackson JB, Feredoes E, Rich AN, Lindner M, Woolgar A. Concurrent neuroimaging and neurostimulation reveals a causal role for dlPFC in coding of task-relevant information. Commun Biol 2021;4:588. 34002006 10.1038/s42003-021-02109-x PMC8128861

[66] Webler RD, Fox J, McTeague LM, Burton PC, Dowdle L, Short EB, et al. DLPFC stimulation alters working memory related activations and performance: an interleaved TMS-fMRI study. Brain Stimulat. 2022;15:823–32. 10.1016/j.brs.2022.05.014 35644517

[67] Tik M, Woletz M, Schuler AL, Vasileiadi M, Cash R, Zalesky A, et al. Acute TMS/fMRI response explains offline TMS network effects – an interleaved TMS-fMRI study. NeuroImage. 2023;267:119833. 36572133 10.1016/j.neuroimage.2022.119833

[68] Hermiller MS, Chen YF, Parrish TB, Voss JL. Evidence for immediate enhancement of hippocampal memory encoding by network-targeted theta-burst stimulation during concurrent fMRI. J. Neurosci. 2020;40:7155–68. 32817326 10.1523/JNEUROSCI.0486-20.2020 PMC7480242

[69] Caparelli EC, Schleyer B, Zhai T, Gu H, Abulseoud OA, Yang Y. High-frequency transcranial magnetic stimulation combined with functional magnetic resonance imaging reveals distinct activation patterns associated with different dorsolateral prefrontal cortex stimulation sites. Neuromodulation Technol Neural Interface. 2022;25:633–43. 10.1016/j.neurom.2022.03.002 35418339

[70] Caparelli EC, Abulseoud OA, Gu H, Zhai T, Schleyer B, Yang Y. Low frequency repetitive transcranial magnetic stimulation to the right dorsolateral prefrontal cortex engages thalamus, striatum, and the default mode network. Front. Neurosci. 2022;16:997259. 36248660 10.3389/fnins.2022.997259 PMC9565480

[71] Patel M, Teferi M, Casalvera A, Lynch KG, Nitchie F, Makhoul W, et al. *Interleaved TMS/fMRI Shows That Threat Decreases dlPFC-Mediated Top-down Regulation of Emotion Processing*. http://medrxiv.org/lookup/doi/10.1101/2023.11.11.23298414 (2023). 10.1101/2023.11.11.23298414 10.1038/s44277-024-00007-8 PMC12624932 41361047

