# Supplementary material for: Interleaved TMS/fMRI shows that threat decreases dlPFC-mediated top-down regulation of emotion processing
Source: NPP Digit Psychiatry Neurosci. 2024 Apr 24;2:6. doi: 10.1038/s44277-024-00007-8 (PMC12624932; doi:10.1038/s44277-024-00007-8)
Supplement: Supplementary file 1 — Supplemental Material [file 44277_2024_7_MOESM1_ESM.docx]

**Title**: Interleaved TMS/fMRI shows that threat decreases dlPFC-mediated top-down regulation of emotion processing.

**Corresponding Author:**

Nicholas Balderston

Center for Neuromodulation in Depression and Stress

3700 Hamilton Walk, Richards D302

Philadelphia, PA, 19104

Phone: 215-746-3058

Fax: 215-573-8556

[nicholas.balderston@pennmedicine.upenn.edu](mailto:nicholas.balderston@pennmedicine.upenn.edu)

**Supplemental Methods**

**General Procedure**

**Targeting visit.** During the targeting visit, MRI scans were collected and used to identify the site and orientation of stimulation for the TMS/fMRI session. Participants were escorted to the scanner and given ear plugs, a button box, an emergency squeeze ball, and padding to minimize head movement. Next, we collected a T1, a T2, and a diffusion-weighted scan. Afterward, participants completed 1 run of the Sternberg WM task and 2 resting state runs.

TMS/fMRI visit. During the TMS/fMRI visit, participants were prepped for scanning prior to being escorted to the scanner. Their head was registered with their MRI in Brainsight and their stimulation sites were marked on a swimcap. Afterward, the participants completed the shock workup procedure and were escorted to the scanner. Once prepped for the first TMS/fMRI scan, the coil was positioned over the right dlPFC stimulation site and the articulating arm was supported with additional padding. The participants then completed 2 runs of the NPU task with TMS pulses targeting the right dlPFC. The participant then completed 2 additional NPU runs with the TMS pulses targeting the IPS.

**Target localization**

Data from the Sternberg WM task was used to identify the right dlPFC target coordinates for the TMS/fMRI session ^24,31^. BOLD maps from the retention interval were masked with a function ROI of the right dlPFC. The ROI was obtained from a group-level analysis using a previously collected Sternberg WM dataset ^32,33^. Single participant BOLD activity was contrasted across sort and maintain trials and the coordinates for the peak voxel within this mask was extracted and used as a target. For the control site, we used the group level coordinates for the right intraparietal sulcus, identified in a previous study ^34,35^. We used the Brainsight (Rogue Research Inc, Montreal, Canada) frameless stereotaxic neuronavigation system to mark the target site on a swim cap worn during the TMS/fMRI session.

**Supplemental Results**

**Control site-evoked responses**

We extracted the average dlPFC-targeted TMS-evoked BOLD response during the safe and threat conditions for each of the clusters identified in the whole brain analysis. We then compared these responses using a paired sample t-test. We repeated this process for our control site evoked responses in a subset of individuals who also had data targeting the right IPS (Supplemental Figure 1). To determine whether the differential responses evoked by the dlPFC targeted TMS pulses were specific to dlPFC stimulation, we examined the responses in these same regions following stimulation to a control site (the right IPS) in a subset of participants. None of these regions showed significant differentiation as a function of threat. Indeed, the site with the largest effect size showed a Cohen’s d of 0.22, which would require ~130 participants to have an 80% chance of detecting an effect with a single-tailed paired-sample t-test.


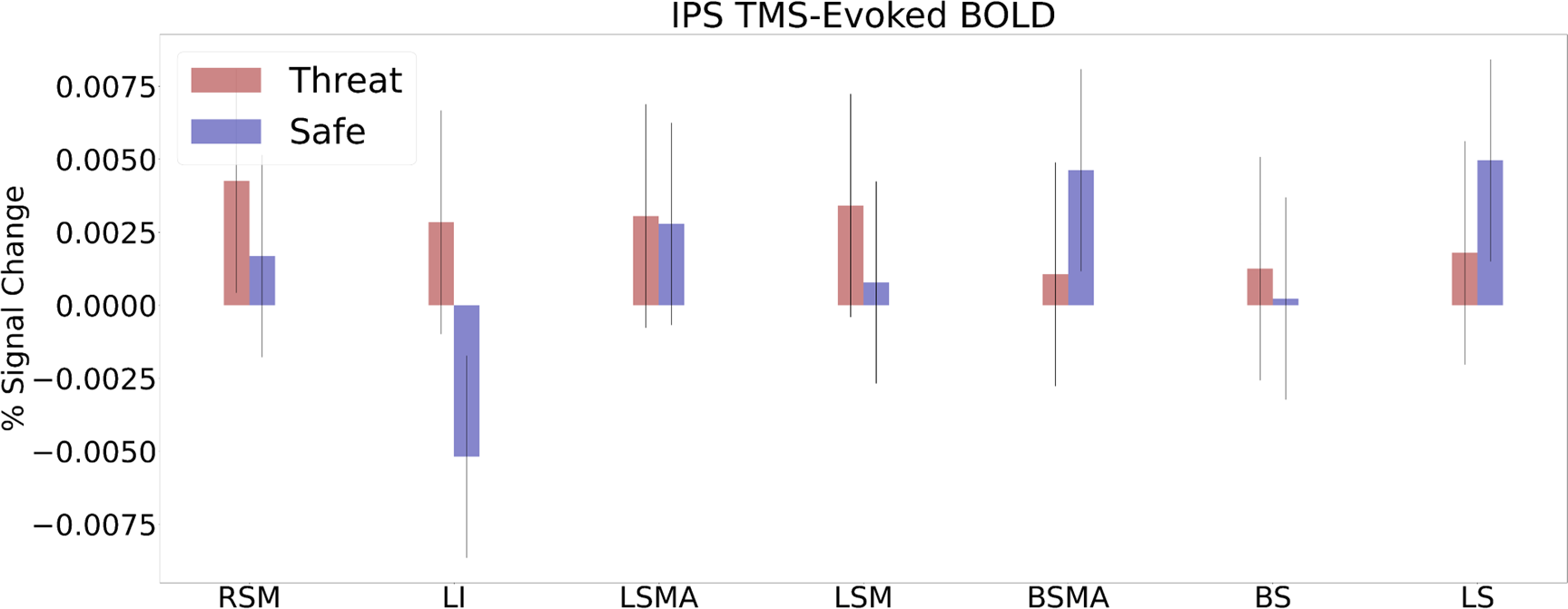


**Supplemental Figure 1. BOLD responses evoked by TMS pulses delivered to a control site.** BOLD responses in the regions from Figure 4 evoked by TMS pulses delivered to a control site, plotted as a function of safe and threat conditions. RSM = Right Sensory/Motor. LI = Left Insula. LSMA = Left SMA. LSM = Left Sensory/Motor. BSMA = Bilateral SMA. BS = Brainstem. LS = Left Sensory. Warm colors represent activations. Cool colors represent deactivations. Bars represent the mean ± SEM.
